# Evaluation of alginate-based coatings enriched with postbiotics from *Bifidobacterium spp*. on the quality and safety of Turkey meat

**DOI:** 10.1038/s41598-025-09913-z

**Published:** 2025-07-02

**Authors:** Emel Cengiz Kaynakci

**Affiliations:** 1 Institute of Health Sciences, Department of Medical Biotechnology, Akdeniz University, 07070 Antalya, Konyaaltı Turkey; 2 Faculty of Health Sciences, Department of Nutrition and Dietetics, Akdeniz University, 07070 Antalya, Konyaaltı Turkey

**Keywords:** Postbiotics, *Bifidobacterium bifidum* BB12, *Bifidobacterium bifidum* DSM 20456, Turkey meat, Alginate, Edible coating, Shelf life, Microbiology, Pathogens

## Abstract

This study evaluated the antimicrobial and antioxidant properties of postbiotics derived from *Bifidobacterium bifidum* DSM 20,456 (b1), *Bifidobacterium bifidum* BB12 (b2), and their combination (bb) in alginate-based edible coatings for fresh turkey breast meat. Coated and uncoated samples were stored at 4 °C for seven days, with microbial and physicochemical analyses conducted at regular intervals (0, 2, 4, 7 days). The postbiotic-enriched coatings exhibited significant antioxidant activity, with total phenolic content (TPC) ranging from 87.13 to 90.89 mg GAE/100 mL and DPPH radical scavenging capacity between 50.28 and 51.56 mg TEAC/100 mL (*p* < 0.05). While TBARS values were lower in postbiotic-treated groups, the differences were not statistically significant (*p* > 0.05). Water holding capacity (WHC) was maintained at higher levels in postbiotic-treated samples throughout storage. Microbial analysis revealed that total aerobic mesophilic and psychrotrophic bacterial counts were not significantly affected (*p* > 0.05), whereas *Listeria monocytogenes* counts were significantly reduced in coated samples (*p* < 0.05), with the bb formulation achieving a 1.62 log₁₀ CFU/g reduction. Fourier Transform Infrared (FTIR) spectroscopy confirmed molecular interactions between postbiotics and the alginate matrix, enhancing coating stability. These findings suggest that postbiotic-alginate coatings effectively inhibit *L. monocytogenes* while maintaining the physicochemical stability of turkey meat, highlighting their potential in poultry preservation. However, further optimization, such as combining postbiotics with additional antimicrobial agents, is needed to enhance overall microbial control.

## Introduction

Turkey meat is a highly nutritious source of protein, rich in essential amino acids, B-complex vitamins including B1, B2, B6, and B12, and minerals such as calcium and phosphorus. It also contains unsaturated fatty acids, making it an attractive option for health-conscious consumers. However, poultry meat, including turkey, is highly perishable due to its susceptibility to microbial contamination and lipid oxidation, which can significantly compromise safety, quality, and shelf life^1,2^.

Foodborne pathogens such as *Listeria monocytogenes*, *Salmonella* spp., and *Staphylococcus aureus* are frequently linked to poultry consumption and remain significant public health concerns^3,4^. In particular, *Listeria monocytogenes* is recognized as a critical foodborne pathogen in the European Union. It can proliferate under refrigerated conditions commonly used in poultry meat storage. The European Food Safety Authority (EFSA) has highlighted the persistent risk posed by *L. monocytogenes* in ready-to-eat meat products, noting its role in severe infections and the challenges associated with its control in the food supply chain^5^. This pathogen’s ability to grow at low temperatures and its resilience against conventional preservation methods underscore the urgent need for novel, natural preservation strategies. These bacteria can proliferate during storage, leading to spoilage and undesirable changes in meat texture, odour, and flavour. Traditional preservation techniques such as refrigeration or chemical preservatives offer temporary solutions but do not align with the growing consumer demand for natural, clean-label products^6,7^.

In recent years, consumer demand for minimally processed and naturally preserved foods has driven the development of innovative packaging technologies. Among these, edible films and coatings derived from natural polymers such as proteins, polysaccharides, and lipids have shown great potential as biodegradable and biocompatible materials that can enhance food safety and quality. These edible coatings provide barriers against moisture loss, oxygen, and microbial contamination, thereby reducing spoilage and extending shelf life. Moreover, edible coatings can be used as carriers for natural antimicrobial agents, including organic acids, chitosan, nisin, and plant extracts, enabling controlled release of bioactive compounds that inhibit pathogenic and spoilage microorganisms. The efficacy of antimicrobial edible coatings depends on complex interactions among the coating matrix, incorporated antimicrobials, target microorganisms, and food components, necessitating careful selection and optimization. Recent studies have demonstrated promising applications of antimicrobial edible films and coatings in meat products, supporting their role as natural preservation strategies^8^.

In this context, postbiotics, non-viable bacterial metabolites produced during probiotic fermentation, have emerged as promising natural preservatives. Compounds such as organic acids, peptides, and bacteriocins derived from postbiotics exhibit antimicrobial and antioxidant properties effective against key pathogens and spoilage bacteria^9–11^. Notably, *Bifidobacterium bifidum* has demonstrated a strong capacity to produce postbiotic substances with activity against *L. monocytogenes* and *S. enterica*^12,13^. Furthermore, recent proteomic studies demonstrate that competition between protective cultures like *Lactococcus lactis* and *L. monocytogenes* can enhance bacteriocin (e.g., nisin) production, strengthening the antimicrobial efficacy of postbiotic-based interventions^14^.

Among postbiotics, biosurfactants have recently gained attention as a novel class of bioactive compounds. Produced by certain beneficial bacteria, biosurfactants possess antimicrobial, emulsifying, and detoxifying properties, contributing to enhanced food safety and quality. Their inclusion in postbiotic formulations offers additional potential for natural preservation and functional food development^15^.

A complementary preservation strategy involves alginate-based edible coatings, which serve as biodegradable, non-toxic films capable of reducing moisture loss, lipid oxidation, and microbial contamination in fresh meat products^11^. These coatings also function as controlled-release systems for bioactive compounds, enhancing the stability and efficacy of incorporated antimicrobials such as postbiotics^16^.

Despite these promising developments, limited research has investigated the combined use of postbiotics and alginate-based edible coatings specifically in fresh turkey meat products. This gap is particularly notable in studies utilizing postbiotics derived from *B. bifidum* strains.

This study aims to evaluate the antimicrobial and antioxidant properties of postbiotics derived from *B. bifidum* DSM 20,456 and BB12 when applied in alginate-based edible coatings for fresh turkey breast meat stored at 4 °C. The effects of these coatings on microbial stability (total bacterial count, *L. monocytogenes* inhibition), physicochemical properties (lipid oxidation, pH, water-holding capacity, color), and shelf life were investigated.

It is hypothesized that alginate-based edible coatings enriched with postbiotics derived from *B. bifidum* DSM 20,456 and BB12 will significantly inhibit *L. monocytogenes*, delay lipid oxidation, and maintain the physicochemical quality of fresh turkey breast meat during cold storage, outperforming uncoated controls in terms of microbial safety and shelf life extension.

## Materials and methods

### Materials

*B. bifidum* DSM 20,456 and *B. bifidum* BB12 strains were sourced from Akdeniz University, Department of Food Engineering, Turkey. on MRS agar (*Lactobacillus* Agar acc. to De Man, Rogosa, and Sharpe), MRS Broth (*Lactobacillus* Broth acc. to DE MAN, ROGOSA and SHARPE), DBRC Agar (DichloranRose Bengal Chloramphenicol), PCA Agar (Casein-peptone Dextrose Yeast Agar) were purchased from BID (Germany). Sodium alginate, calcium chloride, TCA (Trichloroacetic Acid) and TBA (Thiobarbituric Acid) purchased from Merck (Germany). Fresh skinless turkey breast fillets were purchased from Bahar Hindi Meat Industry Co. (Antalya, Turkey) and transported to the laboratory under a cold chain (+ 4 °C).

### Methods

#### Preparation of postbiotics and edible coating

Postbiotics were prepared following the method outlined by^17^ with minor modifications. The cultures were cultivated on MRS agar (*Lactobacillus* Agar acc. to De Man, Rogosa, and Sharpe, BID, Germany) at 37 °C for 24–48 h. The bacterial suspensions were standardized to 12 McFarland, corresponding to approximately 3.6 × 10⁹ CFU/mL, using a densitometer. The cultures were centrifuged at 9,400 × g for 10 min at 4 °C, and the supernatant was filtered through a 0.22 μm syringe filter to obtain the postbiotic extract. The postbiotic solution was adjusted to an 8% concentration (8mL postbiotics/100mL alginate-distilled water) for use in coating applications.

A sodium alginate solution enriched with *B. bifidum* DSM 20,456 and *B. bifidum* BB12 strain postbiotics was used as the coating solution. The formulation was adapted with minor modifications based on^18^. Distilled water was used for preparing all coating solutions. After adding sodium alginate to water, the solutions were stirred and heated at 50 °C for 30 min on a hotplate with a magnetic stirrer (CLS, Turkey) to ensure uniform mixing. After obtaining homogeneous solutions, they were cooled to ambient temperature, and the postbiotic solutions were added at a ratio of 16 mL per 200 mL of alginate solution. Turkey breast pieces, cut into 25 g portions, were immersed in the coating solution for 2 min. To enhance cross-linking in the coatings, the coated samples were subsequently immersed in a 2% calcium chloride solution. After coating, the turkey breast pieces were suspended on hooks and hung to allow excess coating solution to drip off. The samples were then dried in a biological safety cabinet for approximately 30 min.

#### Experimental setup

The Turkey meat was aseptically portioned into 25 g samples using a sterile knife, yielding a total of 100 portions, and stored at 4 °C. Samples were used for microbial and physicochemical analyses. The samples were randomly divided into five groups:

1. Control (unwrapped samples) **(c)**.
2. Samples were coated with only an alginate-based edible coating **(a)**.
3. Samples were coated with an alginate-based edible coating solution enriched with 8% cell-free supernatant of *B. bifidum* DSM 20,456 **(b1)**.
4. Samples were coated with an alginate-based edible coating solution enriched with 8% cell-free supernatant of *B. bifidum* BB12 **(b2)**.
5. Samples were coated with an alginate-based edible coating solution enriched with 4% cell-free supernatant of *B. bifidum* DSM 20,456 and 4% cell-free supernatant of *B. bifidum* BB12 **(bb)**.

#### Preparation of L. monocytogenes pathogenic bacterial inoculum

In this study, *L. monocytogenes* ATCC 19118 was used. Colonies stored at 4–7 °C on blood agar (Merck, 110886, Germany) were transferred using a sterile loop to a selective medium and incubated under optimal conditions for 24–48 h. Following incubation, colonies were suspended in 9 mL of sterile Ringer’s solution (Merck, 115525, Germany), and the inoculum density was adjusted to 0.5 McFarland using a densitometer (Biosan DEN-1B, Biosan, Turkey^19^).

To evaluate the growth of *L. monocytogenes*, the same sample groups were prepared as described above. The inoculum density was standardized to 0.5 McFarland, and 100 µL of the inoculum cocktail was evenly spread over the surface of each 25 g portion using a Drigalski spatula. The samples were then left undisturbed for 10 min to allow bacterial adhesion. After this period, all groups were vacuum-packaged and stored at 4 °C under refrigeration for subsequent analysis. All procedures involving *L. monocytogenes* were carried out under Biosafety Level 2 (BSL-2) conditions in the microbiology laboratory of Akdeniz University, Faculty of Health Sciences. Laboratory work was performed using a Class II biosafety cabinet and standard personal protective equipment (PPE). All biological waste was autoclaved before disposal, following institutional biosafety guidelines.

### Postbiotic analysis

Antioxidant Activity (DPPH), Total Phenolic Content (TPC), Total Flavonoid Content (TFC), and Chemical Composition (GC-MS) of postbiotics.

The antioxidant activity was assessed using DPPH radical scavenging activity, total phenolic content (TPC), and total flavonoid content (TFC) methods. The chemical composition (GC-MS) of postbiotics was conducted by the Food Safety and Agricultural Research Center of Akdeniz University and the Western Mediterranean Agricultural Research Institute Directorate (Antalya, Turkey).

#### Disk diffusion method (DDM) of postbiotics

The antimicrobial efficacy of postbiotics was determined using disk diffusion method (DDM) against foodborne pathogens. The MIC of *B. bifidum* DSM 20,456 and *B. bifidum* BB12 postbiotics was determined against *L. monocytogenes* ATCC 19,118, *Salmonella enterica* ATCC 14,028, *Staphylococcus aureus* ATCC 25,923, *Escherichia coli* ATCC 25,922, and *Bacillus cereus* ATCC 11,778.

The antimicrobial activity of the postbiotics (CFS) was further assessed using the agar disk diffusion method. Pathogens were inoculated onto agar plates at a 6 × 10⁷ log_10_ CFU/mL concentration. Disks impregnated with the postbiotic solution (16 µg/mL) were placed at the center of the inoculated plates. The plates were incubated at 37 ± 1 °C for 24 h, and the diameter of the inhibition zones was measured^18^.

#### Physicochemical analyses of coated Turkey meat during storage

Physicochemical analyses of both control and postbiotic-enriched alginate-coated turkey meat were performed on days 0, 2, 4, and 7 of refrigerated storage at 4 °C. Each analysis was conducted in triplicate.

##### Thiobarbituric acid reactive substances (TBARS) analysis

Lipid oxidation in postbiotic-enriched alginate-coated turkey breast samples was evaluated using the TBARS method described by^20^. Results were calculated using a standard curve prepared from 1,1,3,3-tetraethoxypropane (TEP) and expressed as µmol MDA per kg meat. Two grams of each sample were homogenized with 2 mL of trichloroacetic acid (TCA) solution for 15–20 s, filtered through Whatman No. 1 filter paper, and reacted with 1 mL of thiobarbituric acid (TBA) solution. The mixture was incubated at 100 °C for 40 min in a water bath (BM 302, Nüve), cooled to room temperature, centrifuged at 4,100 g for 10 min (F800, Nüve), and the absorbance of the supernatant was measured at 532 nm.

##### pH measurement

The pH of turkey breast samples was measured using a Hanna HI981036 meat pH meter, specifically designed for meat and meat products^21^.

##### Color evaluation

The surface color of the coated turkey samples was measured at four points using a spectrocolorimeter (LS172, China) calibrated with a white tile. L*, a*, and b* values were recorded, where L* indicates lightness (0–100), a* represents redness or greenness, and b* measures yellowness or blueness^21^.

#### Water holding capacity (WHC)

$$\:WHC\left(\%\right) = \left(\frac{W_i - W_f}{W_i}\right)\times100$$

The WHC was determied using the centrifugation method as described by^18^, with slight modifications. Briefly, 5 g of turkey breast meat was placed in pre-weighed centrifuge tubes and centrifuged at 4,000×g for 10 min at 4 °C (Nuve, Germany). After centrifugation, the supernatant was carefully removed, and the remaining meat sample was reweighed. The WHC was calculated using the following equation:

Wi​; refers to the initial weight of the sample before the centrifugation or pressing procedure. Wf; refers to the final weight of the sample after the removal of released water. This method evaluates the ability of the meat to retain its water content under mechanical stress, providing insights into the effects of postbiotic-alginate coatings on meat juiciness and textural integrity during storage.

### Microbial analysis

Microbial counts were determined by using a stomacher by homogenizing 25 g samples in 225 mL of 0.1% Maximum Recovery Diluent (MRD, Merck, Germany). Serial dilutions up to 10⁻⁶ were prepared, and plating was performed using the spread plate method. Plate Count Agar was incubated at 37 °C for 24–48 h for total viable counts and at 7 °C for 10 days for psychrophilic counts. LAB counts were performed on MRS agar (BID, Germany) under anaerobic conditions at 37 °C for 2 days. For mold and yeast counts, Dichloran Rose Bengal Chloramphenicol agar (DRBC, BID, Germany) was incubated at 25 °C for 3 days^13^. All results were expressed as log₁₀ CFU/mL. Analyses were conducted on days 0, 2, 4, and 7 of storage.

#### Fourier transform infrared spectroscopy (FTIR) analysis

A deepFourier transform infrared spectrophotometer (ATR-FTIR; Varian 1,000 model) was used to examine the chemical bonds of alginate and the postbiotic-enriched alginate coating. FTIR analysis was conducted at 4000–400 cm^−1^ (wavelength), also known as mid-infrared^22^.

#### Statistical analysis

All experiments were conducted in triplicate, and data were expressed as mean ± standard deviation (SD). Statistical analysis was performed using SPSS 23.0 (SPSS Inc., Chicago, IL, USA) for physicochemical and antioxidant analyses, and GraphPad Prism 9 (Dotmatics Electronics, USA) for microbial counts. Prior to analysis, data were assessed for normality using the Shapiro–Wilk test and for homogeneity of variances using Levene’s test. For microbial counts (log₁₀ CFU/mL), which did not meet normality assumptions, a non-parametric Kruskal–Wallis test was used to compare group differences, followed by Dunn’s multiple comparison test where significant differences were observed (*p* < 0.05). Microbial data were visualized using GraphPad Prism. For physicochemical properties (pH, TBARS, WHC, color parameters) and antioxidant activity (DPPH, TPC, TFC), a one-way ANOVA was applied to assess group differences. When significant effects were found, Tukey’s post hoc test was performed for pairwise comparisons (*p* < 0.05). All graphs and statistical outputs were generated using SPSS 23.0.

## Results and discussion

### Antimicrobial activity

*B. bifidum* DSM 20,456 and BB12 postbiotics were tested for antimicrobial activity using the Disk Diffusion Method (DDM) (Table 1).

**Table 1:** Disc diffusion (DDF, mm) values of postbiotics against foodborne pathogens.

	Listeria monocytogenes ATCC 19,118	Salmonella enterica ATCC 14,028	Staphylococcus aureus ATCC 25,923	Escherichia coli ATCC 25,922	Bacillus cereus ATCC 11,778
*B. bifidum* DSM 20,456	13.1 ± 0.02	nd	11.5 ± 2.12	14.30 ± 0.20	nd
*B. bifidum* BB12	14.00 ± 1.41	nd	15.00 ± 1.41	12.50 ± 0.71	nd

Disc diffusion (DDF) values are represented as the diameter (mm) of the inhibition zone, nd: Non-detected.

*B. bifidum* BB12 showed the strongest inhibition against *S. aureus* (15.00 ± 1.41 mm) and *L. monocytogenes* (14.00 ± 1.41 mm), while *B. bifidum* DSM 20,456 had a higher effect on *E. coli* (14.30 ± 0.20 mm) compared to BB12 (12.50 ± 0.71 mm). No inhibition was observed against *Salmonella enterica* and *Bacillus cereus.*

Keykhosravy et al. (2020)^23^ reported stronger inhibition of *L. monocytogenes* (16.66 mm) and *S. enteritidis* (18.33 mm) using chitosan nanoemulsions with essential oils. Similarly, Pereira et al. (2018)^10^ found that whey protein coatings with *Bifidobacterium* postbiotics inhibited *Staphylococcus spp.*,* Pseudomonas spp*., and *Enterobacteriaceae*, with varying efficacy. The lack of inhibition against *S. enterica* and *B. cereus* supports previous findings that Gram-negative bacteria resist postbiotics due to their outer membrane. Akman et al. (2023)^24^ also noted that *L. plantarum* postbiotics had limited effects on Gram-negative bacteria, requiring additional antimicrobials.

### Antioxidant activity, total phenolic and flavonoid contents, and chemical composition (GC-MS) of postbiotics

The antioxidant activity, total phenolic (TPC), and flavonoid (TFC) contents of postbiotics are shown in Table 2. The highest DPPH radical scavenging activity was observed in the bb group (51.56 ± 1.63 mg TEAC/100 mL), which also had the highest TPC (90.89 ± 1.63 mg GAE/100 mL) and TFC (21.34 ± 0.96 mg CE/100 mL). These findings align with^2,25^, who reported strong antioxidant activity in *L. rhamnosus*, *L. reuteri*, and *L. paracasei* postbiotics, linking polyphenols to antioxidant effects. A strong correlation between DPPH activity and TPC/TFC values supports the role of phenolic compounds in radical scavenging, as noted by^9^. Additionally, Sriwattanachai et al. (2018)^26^ highlighted that postbiotic phenolics improve microbial stability and extend shelf life.

**Table 2:** DPPH, total phenolic content (TFC) and total flavonoid content (TFC) of postbiotics.

	b1	b2	bb
DPPH (mg TEAC/100 mL)	50.28 ± 0.20c	51.05 ± 0.68b	51.56 ± 1.63a
Total phenolic content (mg GAE/100 mL)	87.13 ± 2.24c	88.24 ± 1.65b	90.89 ± 1.63a
Total flavonoid content (mg CE/100 mL)	24.20 ± 0.25a	18.83 ± 2.21c	21.34 ± 0.96b

^***a−*c**^: The mean values with different letters are significantly different (*p* < 0.05) **b1**, *Bifidobacterium bifidium* DSM postbiotic; **b2**, *Bifidobacterium bifidium* BB12 postbiotic; **bb**, *B. bifidium* BB12 postbiotic + *B. bifidium* DSM postbiotic (1:1).

Table 3 details the volatile compounds identified in postbiotic coatings (b1, b2, bb), which vary depending on the microbial strain and fermentation/coating conditions. A dominant presence of long-chain alkanes such as n-tetradecane, n-hexadecane, and n-heptadecane was observed across all samples. These compounds have been associated with improved oxidative stability in lipid-rich food systems due to their hydrophobic nature and structural inertness, which may slow down lipid peroxidation and oxygen diffusion into the matrix^27^. Monoterpenes like 1,8-cineole and α-thujene, identified predominantly in b1 and b2, are well-known for their broad-spectrum antimicrobial effects. Their mechanism is often attributed to membrane disruption and interference with microbial respiration pathways, contributing to the inhibitory effects seen on *L. monocytogenes* in this study^21^. Similarly, compounds such as α-pinene, camphene, and trans-2-heptenal are metabolic byproducts from microbial fermentation, and their presence can serve as biomarkers for active secondary metabolism or stress adaptation pathways in probiotic strains^3,4^. Benzaldehyde, detected in b1 and b2, is another notable volatile with reported antibacterial and antifungal activity. It is generally formed through amino acid degradation or fermentation and may contribute synergistically to the observed antimicrobial profile^26^.

Other lipid-derived volatiles, such as 2,2,4,6-pentamethylheptane and 2,4-heptadienal, may result from oxidative degradation of fatty acids during fermentation, and their levels can be indicative of metabolic differences between strains^27^. These compounds also play a role in the sensory perception of meat products, possibly contributing to aroma and flavor complexity^7^.

A particularly interesting finding is the detection of phytane, a diterpenoid with known antioxidant activity. Its presence in relatively high amounts in all groups (especially b2) may explain part of the oxidative stability observed in WHC and TBARS tests, even in the absence of statistically significant differences^9^. Phytane has also been implicated in enhancing shelf-life and maintaining sensory qualities in meat analogues and emulsified systems^9^. Moreover, volatiles derived from lactic acid bacteria have been shown to possess antioxidant properties, further supporting their functional role in postbiotic formulations^28^.

Together, these volatile compounds suggest a multifaceted bioactivity of the postbiotic formulations, involving antimicrobial, antioxidant, and possibly flavor-enhancing roles. However, it is important to acknowledge that no direct bioassays were conducted on isolated volatiles in the present study. As recommended in prior work, future studies should explore structure–function relationships more precisely by correlating specific volatiles with antimicrobial and antioxidant performance using fractionation and targeted assays^26^.

**Table 3:** Volatile compounds of postbiotics (%).

	Compounds (%)	Groups		
		b1	b2	bb
1	α-Thujene	0.71	0.10	nd
2	α-pinene	nd	nd	0.11
3	Camphene	nd	nd	0.02
4	Trans-2- heptenal	nd	nd	0.20
5	1,1-diethoxyisopentane	nd	nd	0.06
6	2- α-pinene	nd	nd	0.03
7	Benzaldehyde	0.02	0.09	nd
8	2,2,4,6- pentamethylheptane	0.11	0.14	0.12
9	2,4-heptadienal	0.06	0.09	0.10
10	2,6-dimethylnoname	0.20	0.27	0.21
11	4,6-dimethylundecane	0.36	0.49	0.38
12	1,8-cineole (eucalyptol)	0.38	0.49	0.38
13	n-dodecane	1.96	2.54	2.04
14	4,7-dimethyllundecane	0.49	0.66	0.51
15	1,1- diethoxyhexane	nd	nd	nd
16	2,7,10-trimethyldodecane	0.59	0.78	0.61
17	n-tetradecane	12.36	6.61	19.24
18	4-methyltetradecane	2.00	2.33	2.04
19	n-hexadecane	8.46	10.06	8.49
20	n-heptadecane	12.82	14.95	12.76
21	Phytane	8.59	11.71	9.95
22	n-eicosane	3.90	4.44	3.69
23	n-heneicosane	7.14	7.15	6.46
24	1-monopalmitin (Dihydroxypropyl hexadecanoate)	34.11	30.36	26.67
	Other compounds	5.74	6.74	5.82
	Total	100	100	100

### Effect of Bifidobacterium spp. Postbiotic-Alginate coating on pH, TBARS, and water holding capacity (WHC)

*Bifidobacterium* spp. postbiotic-alginate coatings were evaluated for their effects on pH, lipid oxidation (TBARS), and water-holding capacity (WHC) in turkey breast meat during storage (Table 4). Meat pH influences quality and microbial stability. The control group showed a gradual decline from 5.71 ± 0.09 (day 0) to 5.48 ± 0.02 (day 7), remaining within the normal range. The bb group had the highest initial pH (5.84 ± 0.01) and maintained significantly higher values until day 4 before dropping to 5.48 ± 0.06 on day 7. The pH decrease across all groups was attributed to postbiotic metabolic activity, leading to lactic acid and short-chain fatty acid accumulation, enhancing microbial inhibition^17^. These findings align with^19^, who reported that *Pediococcus acidilactici* postbiotics affected pH differently depending on meat type.

**Table 4:** Effects of Postbiotic-Enriched alginate coatings in pH, lipid oxidation (TBARS), and Water-Holding capacity (WHC) of fresh Turkey meat during Storage.

	Groups	d0	d2	d4	d7
pH	Control	5.71 ± 0.09a^AB^	5.68 ± 0.03a^A^	5.66 ± 0.03a^A^	5.48 ± 0.02b^A^
	a	5.50 ± 0.07b^C^	5.60 ± 0.02a^B^	5.45 ± 0.04b^B^	5.43 ± 0.01b^AB^
	b1	5.48 ± 0.07bc^C^	5.61 ± 0.03a^B^	5.51 ± 0.02b^B^	5.43 ± 0.03c^AB^
	b2	5.66 ± 0.05a^B^	5.60 ± 0.03a^B^	5.50 ± 0.04b^B^	5.38 ± 0.02c^B^
	bb	5.84 ± 0.01a^A^	5.68 ± 0.02b^A^	5.52 ± 0.05c^B^	5.48 ± 0.06c^A^
TBARS (malonaldehyde µmol/kg)	Control	0.41 ± 0.13	0.46 ± 0.03	0.86 ± 0.06	1.54 ± 0.59
	a	0.45 ± 0.10	0.37 ± 0.06	0.67 ± 0.50	1.08 ± 0.91
	b1	0.41 ± 0.02	0.33 ± 0.12	0.65 ± 0.75	0.93 ± 0.70
	b2	0.56 ± 0.02	0.43 ± 0.04	0.86 ± 1.02	0.98 ± 0.94
	bb	0.60 ± 0.07	0.37 ± 0.06	0.72 ± 1.24	1.03 ± 0.85
WHC	Control	91.98 ± 1,16a	85.76 ± 1.06b	87.44 ± 0.46ab	89.19 ± 1.17ab
	a	92.39 ± 0.04	89.90 ± 1.05	87.20 ± 1.71	89.18 ± 3.14
	b1	90.58 ± 0.33ab	86.12 ± 1.37b	88.35 ± 1.34ab	92.06 ± 1.38a
	b2	95.04 ± 0.76	86.13 ± 4.84	90.47 ± 1.57	89.60 ± 2.90
	bb	92.63 ± 4.21	90.41 ± 1.12	91.83 ± 1.35	93.04 ± 0.62

Values are expressed as mean ± standard deviation. Different lowercase letters in the same row indicate a significant difference between treatments on the same day according to Tukey’s test (*p* < 0.05). Different capital letters in the same column indicate a significant difference between days within the same treatment according to Tukey’s test (*p* < 0.05). Letters are only assigned to values where significant differences were observed.

TBARS values increased over storage, with the control group reaching the highest value (1.54 ± 0.59 µmol MDA/kg) on day 7. Among postbiotic treated samples, b1 had the lowest TBARS (0.93 ± 0.70 µmol MDA/kg), followed by b2 (0.98 ± 0.94 µmol MDA/kg) and bb (1.03 ± 0.85 µmol MDA/kg), though differences were not statistically significant (*p* > 0.05) (Table 4.). The correlation between high antioxidant activity (DPPH, TPC, and TFC) and lower lipid oxidation suggests that postbiotic-derived bioactive compounds contribute to oxidative stability. Similar effects were reported by^29,30^. Humam et al. (2020)^30^ reported similar findings, showing that *L. salivarius* and *L. plantarum* postbiotics reduced TBARS values in meat due to antioxidant enzyme activity. Additionally, chitosan coatings enriched with postbiotics inhibited lipid oxidation, though their effectiveness may depend on concentration^29^.

WHC influences meat texture and juiciness. Across all groups, WHC ranged from 85.76 to 95.04%, with b1 and bb showing higher values at later storage stages, while the control group declined over time. Although differences were observed, they were not statistically significant (*p* > 0.05). The WHC stabilization in postbiotic-treated groups suggests potential interactions between postbiotics and muscle proteins or protective barrier effects, as noted by^31^.

However, despite the observed trends, TBARS differences were not statistically significant, possibly due to limited bioavailability, compound instability, or entrapment of postbiotic-derived antioxidants within the alginate matrix. Similar findings have been reported in the literature. For instance, Cazón et al. (2019)^32^ demonstrated that although alginate-based films enriched with antioxidant compounds showed strong radical scavenging activity, their effectiveness in lipid oxidation inhibition was limited due to matrix interactions and reduced compound mobility. Therefore, the non-significant TBARS inhibition in our study may be attributed to structural characteristics of the alginate coating affecting compound release and stability during storage.

### Effect of Postbiotic-Alginate coatings on the color (L, a*, b*) of Turkey breast meat

The effects of *Bifidobacterium* spp. postbiotic-alginate coatings on pH, TBARS and WHC in turkey breast meat during storage were evaluated (Table 5).

**Table 5:** Effects of postbiotics derived from *Bifidobacterium spp.* On color (L*, a*, and b* color values).

	Groups	D0	d2	d4	d7
L	Control	38.76 ± 4.12	42.16 ± 3.07	37.86 ± 5.43	40.23 ± 1.19
	a	40.73 ± 5.41	42.74 ± 3.40	45.38 ± 5.49	49.22 ± 2.05
	b1	42.20 ± 3.09ab	47.00 ± 2.29a	35.02 ± 4.46b	35.02 ± 4.46ab
	b2	36.48 ± 4.11b	47.17 ± 1.76a	45.91 ± 5.26ab	51.02 ± 6.84a
	bb	38.93 ± 2.03	44.94 ± 3.72	41.14 ± 6.20	48.30 ± 10.91
a*	Control	1.04 ± 1.28a^AB^	1.06 ± 0.91a^AB^	−0.61 ± 1.44a^A^	−0.35 ± 0.96a^A^
	a	2.27 ± 0.50a^A^	2.56 ± 0.54a^A^	−1.41 ± 0.58c^A^	0.40 ± 0.48b^A^
	b1	0.30 ± 0.73a^AB^	1.45 ± 1.17a^AB^	0.58 ± 1.18a^A^	0.65 ± 1.54a^A^
	b2	1.14 ± 1.07a^AB^	0.66 ± 0.13ab^B^	−0.56 ± 0.73b^A^	0.36 ± 0.54b^A^
	bb	0.08 ± 0.77a^B^	0.38 ± 0.97a^B^	−0.18 ± 1.12a^A^	−0.03 ± 0.71a^A^
b*	Control	2.70 ± 1.22a^B^	4.55 ± 1.16a^B^	2.85 ± 2.46a^A^	1.77 ± 0.62a^A^
	a	4.61 ± 1.79a^A^	5.47 ± 2.22a^B^	2.56 ± 0.53b^A^	2.09 ± 1.03b^A^
	b1	3.54 ± 0.45ab^AB^	5.56 ± 1.02a^AB^	2.91 ± 0.88b^A^	3.09 ± 2.00ab^A^
	b2	3.99 ± 1.54a^AB^	4.04 ± 0.33a^A^	2.11 ± 0.89ab^A^	1.38 ± 0.10b^A^
	bb	3.28 ± 0.47a^B^	3.32 ± 1.28a^B^	2.86 ± 0.81a^A^	2.24 ± 1.02a^A^

Values are expressed as mean standard deviation. Different lowercase line letters in the same indicate a significant difference between the days by Tukey’s test (*p* < 0.05). Different capital letters on the same column indicate a significant difference between treatments by Tukey’s test (*p* < 0.05) Letters are only assigned to values where significant differences were observed.

Muscle pH affects meat quality and microbial stability. Ideally, meat reaches a final pH of 5.8–5.4 within 24 h of slaughter, while a pH above 6.0 indicates quality problems. High pH reduces oxygenation, which affects colour, tenderness and water retention, while increasing the risk of spoilage (de Lima et al. 2014)^33^. In this study, the control group showed a gradual decrease in pH from 5.71 ± 0.09 (day 0) to 5.48 ± 0.02 (day 7), which remained within the expected range. The bb group, with the highest initial pH (5.84 ± 0.01), maintained significantly higher values until day 4, before decreasing to 5.48 ± 0.06 on day 7. The b1 and b2 groups showed a more stable decrease from 5.48 ± 0.07 to 5.38 ± 0.02 by day 7. These results suggest that postbiotic coatings influence pH stability, probably due to the accumulation of lactic acid and short-chain fatty acids, which enhance microbial inhibition^25^. Incili et al. (2021a)^19^ found that *P. acidilactici* postbiotics did not significantly alter the pH of chicken breasts, but lowered the pH of drumsticks for up to six days. Lipid oxidation, a key factor in meat deterioration, was assessed using TBARS values. TBARS values increased over storage, with the control group reaching the highest value (1.54 ± 0.59 µmol MDA/kg) at day 7. Among postbiotic-treated samples, b1 had the lowest TBARS (0.93 ± 0.70 µmol MDA/kg), followed by b2 (0.98 ± 0.94 µmol MDA/kg) and bb (1.03 ± 0.85 µmol MDA/kg), though differences were not statistically significant (*p* > 0.05). Higher antioxidant activity (DPPH, TPC, TFC) correlated with lower lipid oxidation, suggesting that postbiotic bioactive compounds contribute to oxidative stability. Moradi et al. (2017)^29^ and Humam et al. (2020)^30^ reported similar findings, showing that *L. salivarius* and *L. plantarum* postbiotics reduced TBARS values in meat due to antioxidant enzyme activity. Additionally, chitosan coatings enriched with postbiotics inhibited lipid oxidation, though their effectiveness may depend on concentration.

pH also influences oxidative stability, as higher pH reduces pro-oxidant iron availability and stabilises muscle proteins, slowing lipid oxidation^34^. In this study, pH differed significantly between treatments (*p* < 0.05) and decreased over time. However, no direct correlation was found between pH and TBARS levels, suggesting that postbiotic bioactive compounds play a greater role in oxidative stabilisation. Another possible mechanism is the oxygen barrier effect of edible coatings. Namratha et al. (2020)^35^ reported that pectin-alginate-casein coatings reduced lipid oxidation by limiting oxygen diffusion. Similarly, Campaniello et al. (2020)^13^ found that *Bifidobacterium animalis* in alginate-gelatine coatings reduced lipid oxidation, probably through a combination of barrier effects and release of antioxidant metabolites. While the postbiotic treated groups showed lower TBARS levels, the lack of statistical significance (*p* > 0.05) suggests that the effects may be dependent on concentration or formulation specific factors. Previous studies suggest that higher concentrations of postbiotics produce stronger antioxidant effects^29^, which may explain the variation in results.

Water holding capacity (WHC) affects meat quality, texture and juiciness. WHC was measured over seven days to evaluate the effects of *Bifidobacterium* spp. postbiotic treatments. Although numerical variations were observed, no statistically significant differences were found between the groups (*p* > 0.05). WHC ranged from 85.76 to 95.04%, with b1 and bb showing higher values at later stages, while the control group showed a decrease. b1 reached the highest WHC on day 7, suggesting a delayed postbiotic effect.

Previous studies have shown mixed results. Fang et al. (2024)^34^ found no significant effects of *Bacillus subtilis* postbiotics on WHC, while Humam et al. (2020)^30^ reported reduced drip loss but inconsistent changes in WHC. Although significant pH differences were observed (*p* < 0.05), WHC was not statistically different between treatments (*p* > 0.05) suggesting that WHC depends on factors beyond pH, such as muscle structure and postbiotic interactions. Malvano et al. (2022)^31^ demonstrated that alginate-based coatings improved WHC by reducing water loss, which may explain the stability of WHC in b1 and bb.

### Effects of Postbiotic-Alginate coatings on the microbial stability of Turkey breast meat

**Fig. 1:**
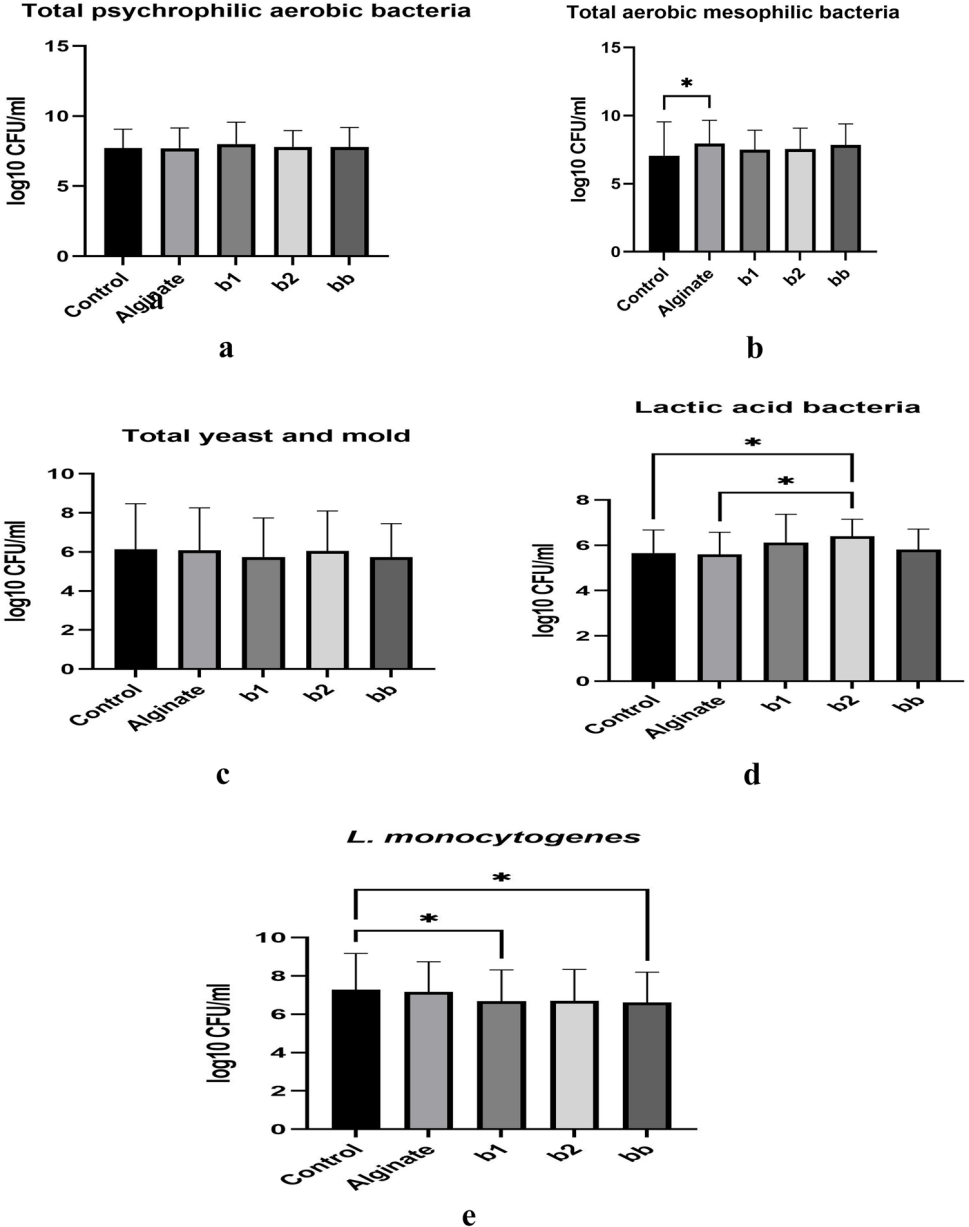
(**a**) The mean number of PAB in turkey breast meat coated with postbiotics (log_10_ CFU/ml).*A significant difference was found at the *p* < 0.05 level according to the Kruskal-Wallis test. **Control**, without edible coating; **alginate**, only sodium alginate coated; **b1**, sodium alginate with *Bifidobacterium bifidum* DSM postbiotic coated group; **b2**, sodium alginate with *Bifidobacterium bifidium* BB12 postbiotic coated group; **bb**, sodium alginate with *Bifidobacterium bifidium* BB12 postbiotic + *Bifidobacterium bifidium* DSM postbiotic coated group. (**b**) The mean number of TMAB in turkey breast meat coated with postbiotics (log_10_ CFU/ml) *A significant difference was found at the *p* < 0.05 level according to the Kruskal-Wallis test. **Control**, without edible coating; **alginate**, only sodium alginate coated; **b1**, sodium alginate with *Bifidobacterium bifidum* DSM postbiotic coated group; **b2**, sodium alginate with *Bifidobacterium bifidium* BB12 postbiotic coated group; **bb**, sodium alginate with *Bifidobacterium bifidium* BB12 postbiotic + *Bifidobacterium bifidium* DSM postbiotic coated group. (**c**) The mean number of yeast molds in turkey breast meat coated with postbiotics (log_10_ CFU/ml) *A significant difference was found at the *p* < 0.05 level according to the Kruskal-Wallis test. **Control**, without edible coating; **alginate**, only sodium alginate coated; **b1**, sodium alginate with *Bifidobacterium bifidum* DSM postbiotic coated group; **b2**, sodium alginate with *Bifidobacterium bifidium* BB12 postbiotic coated group; **bb**, sodium alginate with *Bifidobacterium bifidium* BB12 postbiotic + *Bifidobacterium bifidium* DSM postbiotic coated group. (**d**) The mean number of LAB in turkey breast meat coated with postbiotics (log_10_ CFU/ml) *A significant difference was found at the *p* < 0.05 level according to the Kruskal-Wallis test. **Control**, without edible coating; **alginate**, only sodium alginate coated; **b1**, sodium alginate with *Bifidobacterium bifidum* DSM postbiotic coated group; **b2**, sodium alginate with *Bifidobacterium bifidium* BB12 postbiotic coated group; **bb**, sodium alginate with *Bifidobacterium bifidium* BB12 postbiotic + *Bifidobacterium bifidium* DSM postbiotic coated group. (**e**) The mean number of total *L. monocytogenes* in turkey breast meat coated with postbiotics (log_10_ CFU/ml).*A significant difference was found at the *p* < 0.05 level according to the Kruskal-Wallis test. **Control**, without edible coating; **alginate**, only sodium alginate coated; **b1**, sodium alginate with *Bifidobacterium bifidum* DSM postbiotic coated group; **b2**, sodium alginate with *Bifidobacterium bifidium* BB12 postbiotic coated group; **bb**, sodium alginate with *Bifidobacterium bifidium* BB12 postbiotic + *Bifidobacterium bifidium* DSM postbiotic coated group.

 Microbial stability is a key factor in meat quality and has a direct impact on food safety, shelf life and spoilage rates. Postbiotic and probiotic-based edible coatings have emerged as innovative strategies to control spoilage microorganisms and pathogenic bacteria through mechanisms such as organic acid production, bacteriocin secretion, oxygen barrier effects and competitive microbial interactions^6^. In this study, *Bifidobacterium* spp. postbiotic-alginate coatings were evaluated for their effects on total aerobic mesophilic bacteria (TAMB), psychrophilic bacteria (PAB), lactic acid bacteria (LAB), yeast, mould and *L. monocytogenes* (Fig. 1a–e)

The results indicate that TAMB levels increased over storage across all treatment groups, with the highest bacterial load observed in the bb group (9.18 log₁₀ CFU/ml at day 7), while b1 exhibited the lowest bacterial growth (8.86 log₁₀ CFU/ml at day 7) (Fig. 1b).

Similarly, PAB increased from an initial range of 6.23–6.51 log₁₀ CFU/ml to a final range of 9.00-9.45 log₁₀ CFU/ml, indicating that postbiotic-alginate coatings had limited inhibitory effects on psychrophilic bacterial growth (Fig. 1a). These findings align with previous research demonstrating that postbiotic coatings delay but do not completely inhibit bacterial proliferation. İncili et al. (2021b)^17^ observed that *P. acidilactici* postbiotic-chitosan coatings reduced TAMB levels by approximately 1.2 log₁₀ CFU/ml compared to untreated samples, extending the microbial stability of frankfurters. Similarly, Abbasi et al. (2023)^1^ found that *Saccharomyces cerevisiae* var. boulardii postbiotic coatings delayed bacterial growth, with final TAMB and PAB counts approximately 0.8 log₁₀ CFU/ml lower than controls. However, despite this observed effect, bacterial growth was not fully inhibited, suggesting that higher concentrations of postbiotics or combinations with antimicrobial agents (e.g., essential oils) may be needed for stronger inhibition, consistent with the findings of^23^.

Yeast and mold counts increased over time, with the lowest levels observed in bb-treated samples (7.71 log₁₀ CFU/ml at day 7), compared to the control (9.05 log₁₀ CFU/ml at day 7) (Fig. 1c.). These results suggest that postbiotic coatings may have some antifungal properties, though their effects were limited. Previous research has reported similar findings, showing that postbiotic-based coatings can partially inhibit fungal proliferation.

Sriwattanachai et al. (2018)^26^ demonstrated that *Lactobacillus plantarum* postbiotic coatings reduced *Penicillium* spp. growth by approximately 1.4 log₁₀ CFU/ml, though complete inhibition was not achieved. Similarly, Abbasi et al. (2023)^1^ found that postbiotic-based coatings lowered fungal counts by 0.9 log₁₀ CFU/ml compared to untreated samples, though their antifungal effects weakened over time. These results indicate that postbiotic-alginate coatings may help delay fungal contamination, but their effects remain limited, reinforcing the need for additional antifungals agents to achieve stronger inhibition.LAB counts increased over storage, with b2 exhibiting the highest final LAB levels (7.16 log₁₀ CFU/ml), followed by b1 (7.03 log₁₀ CFU/ml) and bb (6.80 log₁₀ CFU/ml) (Fig. 1d). These results suggest that postbiotic treatments selectively promoted LAB growth, which is beneficial for microbial stability and meat preservation. This increase in LAB is consistent with previous studies, which report that postbiotic and probiotic coatings can enhance beneficial bacterial populations. Abbasi et al. (2024)^16^ found that *Lactobacillus brevis* postbiotic-based coatings increased LAB counts by 1.5 log₁₀ CFU/ml, contributing to microbial stability in poultry products. Similarly, Namratha et al. (2020)^35^ observed that probiotic-alginate coatings increased LAB populations by 1.2 log₁₀ CFU/ml, reinforcing the role of beneficial microbiota in meat bio preservation. These findings suggest that postbiotic coatings can selectively enhance LAB proliferation, thereby improving microbial stability without the need for synthetic preservatives^6^. *L. monocytogenes* is a major foodborne pathogen, and its control is crucial in meat preservation. The growth of *L. monocytogenes* was evaluated across all groups during refrigerated storage (Fig. 1e). In this study, *L. monocytogenes* counts increased across all groups, with the control group showing the highest bacterial load at 9.35 log₁₀ CFU/ml on day 7. A significant reduction (*p* < 0.05) was observed in the b1 and bb groups compared to the control group, indicating the inhibitory effect of postbiotic-enriched coatings. Specifically, *L. monocytogenes* levels reached 9.35 log₁₀ CFU/ml in the control group, whereas in the bb group, bacterial counts remained at 7.73 log₁₀ CFU/ml, indicating a 1.62 log₁₀ CFU/ml reduction. Similarly, b1 also exhibited significant inhibition (*p* < 0.05), reducing *L. monocytogenes* by 1.26 log₁₀ CFU/ml compared to the control. According to the Turkish Food Codex Microbiological Criteria Regulation (2011)^36^, raw poultry meat must not exceed 7.0 log₁₀ CFU/ml for total aerobic mesophilic bacteria and must be free of *L. monocytogenes* in 25 g samples. Although the postbiotic-alginate coatings reduced microbial counts, the levels observed in this study exceeded these regulatory limits, indicating the need for additional preservation strategies to ensure product safety and compliance with national food safety standards.

The bb treatment exhibited the lowest *L. monocytogenes* count at 7.73 log₁₀ CFU/ml, indicating that postbiotic-alginate coatings provided some inhibition but were not fully effective in controlling pathogen growth (*p* < 0.05). Previous studies have reported similar findings. Pereira et al. (2018)^10^ observed that postbiotic-enriched coatings reduced *L. monocytogenes* by 1.1 log₁₀ CFU/ml compared to untreated controls. Similarly, Campaniello et al. (2020)^10^ demonstrated that *Bifidobacterium animalis* in alginate-gelatin coatings reduced *L. monocytogenes* proliferation by approximately 0.9 log₁₀ CFU/ml. The effect of *L. monocytogenes* inhibition also appears to be strain specific. Moradi et al. (2019)^29^ reported that *Pediococcus acidilactici-*fortified coatings reduced *L. monocytogenes* by 1.5 log₁₀ CFU/ml, a result comparable to the bb group in this study. İncili et al. (2021a)^19^ found that a combination of *Pediococcus acidilactici* postbiotic and chitosan reduced *L. monocytogenes* by 1.5 log₁₀ CFU/ml, comparable to the findings in the bb group. These findings suggest that postbiotic-based coatings can contribute to pathogen control, but additional antimicrobial strategies may be required for full inhibition. The antimicrobial effect of postbiotic-alginate coatings may also be attributed to the formation of a protective barrier that limits bacterial proliferation. Manassi et al. (2022)^37^ noted that postbiotic-functionalized meat coatings provide a dual benefit by both physically restricting microbial contamination and biochemically inhibiting bacterial growth through organic acid release. This aligns with the observed reduction in *L. monocytogenes* in the bb and b1 groups.

### FTIR analysis of Postbiotic-Enriched alginate coatings

The FTIR spectra of postbiotic-enriched alginate coatings revealed significant molecular interactions, particularly in the hydroxyl (-OH) (~ 3,200–3,400 cm⁻¹) and carboxyl (–COO) (~ 1,600 cm⁻¹) regions. These shifts suggest the formation of hydrogen bonds and electrostatic interactions between alginate and postbiotic compounds, which may contribute to the stability of the coating. The observed spectral changes are consistent with previous studies on alginate-based antimicrobial coatings, where the integration of bioactive compounds modified the polymer network and enhanced functionality^33,38,39^. In particular, the 1,500–1,700 cm⁻¹ region, corresponding to amide-related vibrations, showed a marked peak shift in the BB formulation, suggesting stronger interactions between the proteinaceous postbiotic components and the alginate matrix. This phenomenon has been reported in polyphenol-alginate coatings, where bioactive molecules enhance structural integrity through hydrogen bonding and protein-polymer interactions^39^.

**Fig. 2:**
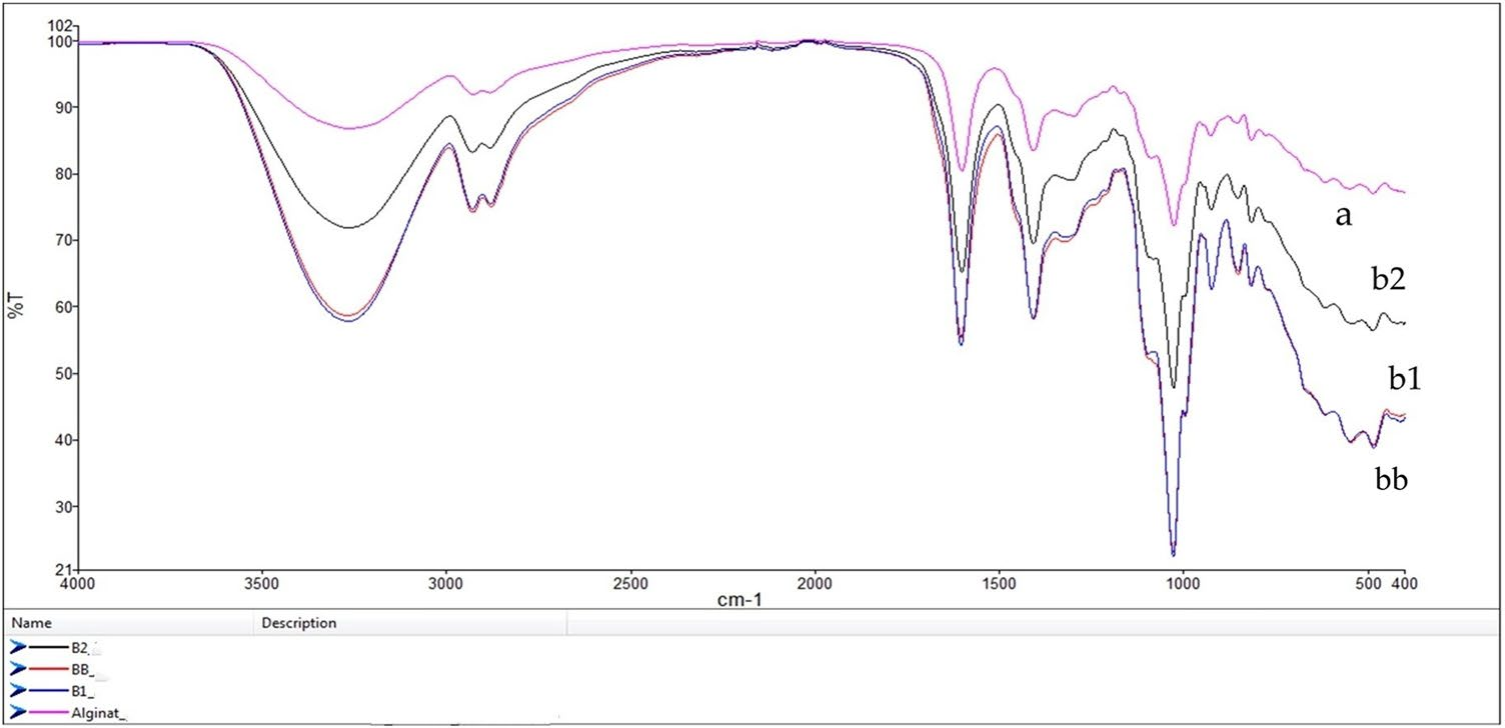
FTR analysis of postbiotic-alginate coating.

Furthermore, increased spectral intensity in the 1,000–1,200 cm⁻¹ range indicates C-O-C stretching vibrations, suggesting enhanced crosslinking and structural reinforcement in postbiotic-enriched coatings. These findings agree with previous research on chitosan-alginate films containing red beetroot anthocyanins, where similar spectral modifications were associated with improved water vapor barrier properties and microbial stability^40^.

Similar structural phenomena were also reported by^41^, who observed that alginate-based microparticles encapsulating *Bifidobacterium animalis* showed specific FTIR shifts related to alginate–protein interactions and suggested their relevance for encapsulation efficiency and matrix stability. Likewise, Cazón et al. (2020)^32^ emphasized that alginate–polymer films’ FTIR spectra can reveal direct evidence of electrostatic interactions and matrix rearrangements when active ingredients like phenolics or postbiotics are added. Moreover, Wang et al. (2024)^42^ highlighted that changes in FTIR peaks correspond to the compatibility and dispersion of bioactives in composite biopolymer films, contributing to their antimicrobial function. While the presence of amide-related peaks (~ 1,540 cm-¹) suggests possible protein-lipid interactions that may contribute to antimicrobial properties, FTIR alone does not confirm bacterial membrane disruption. Previous studies have shown that *Lactiplantibacillus plantarum* postbiotics in sodium alginate films exhibit antibacterial activity, with FTIR peak shifts supporting structural changes^11^. However, additional quantitative techniques such as second derivative FTIR, XRD or thermal analysis (e.g. DSC or TGA) are required to confirm the extent of these molecular interactions and their role in the functionality of the films.

## Conclusions

A total of 24 volatile compounds were identified in the postbiotics derived from B. bifidum DSM 20,456 and BB12. The findings of this study demonstrated that postbiotic-alginate coatings significantly inhibited Listeria monocytogenes growth on fresh turkey meat, achieving a reduction of up to 1.62 log₁₀ CFU/ml in the bb group compared to the control. However, the coatings had a limited effect on total aerobic and psychrotrophic bacterial counts, suggesting that additional antimicrobial agents may be required for broader microbial inhibition. Postbiotic-alginate coatings contributed to maintaining water-holding capacity (WHC) during storage, which is critical for preserving meat quality. While the coatings exhibited antioxidant activity, their effect on lipid oxidation inhibition was moderate and did not reach statistical significance. Fourier Transform Infrared Spectroscopy (FTIR) analysis confirmed molecular interactions between postbiotics and the alginate matrix, suggesting improved structural stability of the coating. The combination of postbiotics and alginate coatings demonstrated a synergistic effect, particularly in controlling *L. monocytogenes*, without significantly altering the pH or color parameters of the turkey meat. These results highlight the potential of postbiotic-enriched edible coatings as a natural intervention for poultry preservation. However, further research is needed to optimize alginate concentration and physicochemical properties (e.g., viscosity, permeability, film thickness), and to characterize the interactions between alginate and postbiotics in terms of release dynamics and barrier effects. These factors may influence the efficacy of microbial inhibition and antioxidant performance. Additionally, although 24 volatile compounds were identified by GC-MS, their individual contributions to antimicrobial activity were not determined. Future studies should investigate the specific mechanisms of action of these compounds using targeted functional assays. Moreover, the present study did not include sensory evaluation, which is a critical factor for consumer acceptance. Future work should incorporate sensory assessments (e.g., texture, odor, and flavor perception) to validate the practical applicability of postbiotic-alginate coatings in meat products. Expanding these studies will provide deeper insights into the bioactive metabolites responsible for antimicrobial and antioxidant effects, ultimately supporting the development of clean-label meat preservation strategies. Additionally, while the study design involved multiple replicates to enhance reliability, a formal statistical power analysis was not conducted. This may limit the interpretation of non-significant outcomes, such as TBARS or total bacterial counts. Future studies should incorporate power analysis to strengthen the statistical robustness and reliability of conclusions. Future work should also consider integrating microbial kinetic models to predict spoilage progression and estimate shelf-life extension under realistic storage scenarios.

## Data Availability

The datasets used and/or analyzed during the current study are available from the corresponding author Emel Kaynakcı on reasonable request via e-mail ekaynakci@akdeniz.edu.tr.
